# Therapeutic Delivery of Tumor Suppressor miRNAs for Breast Cancer Treatment

**DOI:** 10.3390/biology12030467

**Published:** 2023-03-19

**Authors:** Sonali S. Shinde, Sakeel Ahmed, Jonaid Ahmad Malik, Umme Hani, Afreen Khanam, Faisal Ashraf Bhat, Suhail Ahmad Mir, Mohammed Ghazwani, Shadma Wahab, Nazima Haider, Abdulrahman A. Almehizia

**Affiliations:** 1 Department of Chemical Technology, Dr. Babasaheb Ambedkar Marathwada University, Aurangabad 431004, India; 2 Department of Pharmacology and Toxicology, National Institute of Pharmaceutical Education and Research, Ahmedabad 382355, India; 3 Department of Pharmacology and Toxicology, National Institute of Pharmaceutical Education and Research, Guwahati 781101, India; 4 Department of Biomedical Engineering, Indian Institute of Technology, Rupnagar 140001, India; 5 Department of Pharmaceutics, College of Pharmacy, King Khalid University, Abha 62529, Saudi Arabia; 6 Department of Pharmacognosy and Phytochemistry, Jamia Hamdard, New Delhi 110062, India; 7 Department of Pharmacology, Jamia Hamdard, New Delhi 110062, India; 8 Department of Pharmaceutical Sciences, University of Kashmir, Jammu and Kashmir, Hazratbal, Srinagar 190006, India; 9 Department of Pharmacognosy, College of Pharmacy, King Khalid University, Abha 62529, Saudi Arabia; 10 Department of Pathology, College of Medicine, King Khalid University, Abha 62529, Saudi Arabia; 11 Department of Pharmaceutical Chemistry, College of Pharmacy, King Saud University, Riyadh 11451, Saudi Arabia

**Keywords:** breast cancer, miRNAs, multiple drug resistance, nanotechnology

## Abstract

**Simple Summary:**

The most frequent cancer in women is breast cancer (BC), which presents a significant risk to their good health. Over the past few decades, several factors have significantly affected BC progression and treatment. Additionally, microRNAs (miRNAs) that govern several oncogenes and tumor suppressors significantly control the expression networks during BC progression. As a result, miRNA-based therapies that changes these networks may modify the cellular activity to the point where they can treat BC. However, the most substantial challenges in developing such miRNA therapies are the stability and efficacy of their delivery systems. A comprehensive update describing various tumor suppressor miRNAs (TS miRNAs) in BC and their various delivery systems are discussed in this review.

**Abstract:**

The death rate from breast cancer (BC) has dropped due to early detection and sophisticated therapeutic options, yet drug resistance and relapse remain barriers to effective, systematic treatment. Multiple mechanisms underlying miRNAs appear crucial in practically every aspect of cancer progression, including carcinogenesis, metastasis, and drug resistance, as evidenced by the elucidation of drug resistance. Non-coding RNAs called microRNAs (miRNAs) attach to complementary messenger RNAs and degrade them to inhibit the expression and translation to proteins. Evidence suggests that miRNAs play a vital role in developing numerous diseases, including cancer. They affect genes critical for cellular differentiation, proliferation, apoptosis, and metabolism. Recently studies have demonstrated that miRNAs serve as valuable biomarkers for BC. The contrast in the expression of miRNAs in normal tissue cells and tumors suggest that miRNAs are involved in breast cancer. The important aspect behind cancer etiology is the deregulation of miRNAs that can specifically influence cellular physiology. The main objective of this review is to emphasize the role and therapeutic capacity of tumor suppressor miRNAs in BC and the advancement in the delivery system that can deliver miRNAs specifically to cancerous cells. Various approaches are used to deliver these miRNAs to the cancer cells with the help of carrier molecules, like nanoparticles, poly D, L-lactic-co-glycolic acid (PLGA) particles, PEI polymers, modified extracellular vesicles, dendrimers, and liposomes. Additionally, we discuss advanced strategies of TS miRNA delivery techniques such as viral delivery, self-assembled RNA-triple-helix hydrogel drug delivery systems, and hyaluronic acid/protamine sulfate inter-polyelectrolyte complexes. Subsequently, we discuss challenges and prospects on TS miRNA therapeutic delivery in BC management so that miRNAs will become a routine technique in developing individualized patient profiles.

## 1. Introduction

BC is a leading cause of morbidity, disability, and mortality in women worldwide and is a severe health risk [1]. For the first time in 2020, BC overtook lung cancer in terms of overall cancer diagnoses. It accounts for 15.5% of cancer-related fatalities worldwide and is the primary cause of cancer deaths in females in 110 countries. With more than 7.7 million women living 5 years after diagnosis, BC is the most common type of cancer [2]. Because BC is prone to recurrence and the spread of metastases to numerous important organs such as the lungs, brain, liver, and bone, it is imperative to comprehend the key players and the molecular mechanisms driving BC metastasis resulting in the patient’s death [3,4]. Despite significant improvements in the early detection and treatment of BC, some patient groups tend to have worse outcomes [5]. Cellular proteins and RNA interact in a complicated fashion for cellular survival, division, and adaptability. Only 5% of the human genome’s function is understood, while the remaining 95% is still under investigation [6].

**Figure 1:**
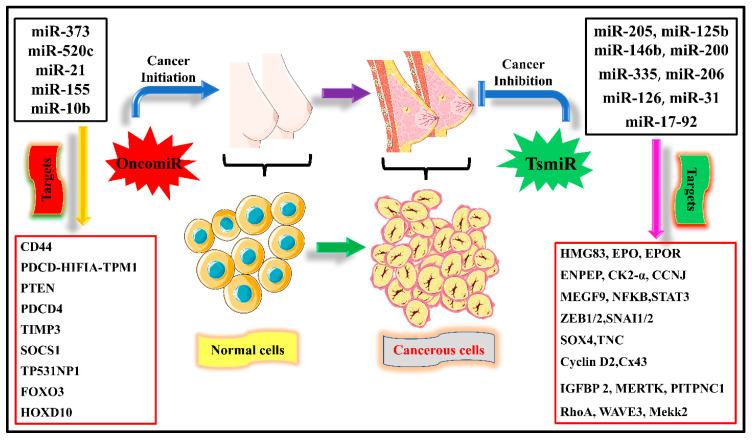
An overview of various miRNAs responsible for initiating cell proliferation and invasion in breast cancer, which are called oncogenic miRNAs (oncomiRs). While miRNAs responsible for tumor suppression are tumor suppressor miRNAs (TS miR), whose main function is the inhibition of migration, invasion, proliferation, and apoptosis. Different miRNAs have different targets for their activity in suppressing tumor growth.

Micro ribonucleic acids (miRNAs) are originally short non-coding RNAs made up of 22 nucleotides that allow gene expression by binding with target mRNAs’ 3′-untranslated regions [7]. They act as negative transcriptional regulators and control various biological processes such as cell survival, apoptosis, metastasis, and proliferation, and tumor miRNAs bind to complementary mRNA sequences and modify them post-translationally via repression, degradation, and silence. Transcription of RNA polymerase II, which folds back to generate another micro mRNA, has a distinctive hairpin form, and this structure is created using longer hairpin structures [8,9,10]. It is known that miRNAs are necessary for several biological activities, including animal development and reproduction, differentiation, maturation, and metabolism [11]. Many metabolic diseases are linked to modifications in miRNA expression [12]. Various diseases may also have altered expression, with significant changes in tumor tissues [13]. The biomolecular diagnosis of cancer has been connected to the miRNA profile [14]. Thus the use of miRNA in cancer diagnostics and treatment is increasing [15]. By interacting with target cell genes, miRNAs control tumor growth and metastasis. Exosomal shuttle small RNAs mediate cell-to-cell communication and cancer metastasis [16]. In altering these intracellular signaling processes, miRNAs can behave as tumor oncogenes or suppressors [17,18]. As shown in Figure 1, excessive expression of miRNAs is already known to impact tumor cell proliferation and development significantly by activating various oncogenic signaling pathways. The oncogenic activity of miRNAs is mediated by targeting numerous tumor suppressors, which causes tumor growth and metastasis in people with BC.

The current study covers the roles played by different tumor suppressor (TS) miRNAs in controlling biological processes, molecular mechanisms, treatment resistance, and various therapeutic delivery techniques of TS miRNA in managing BC. The approach taken in writing this review is based on searching different scientific engines and databases for all kinds of English-language reviews published online during the last ten years, including ScienceDirect, PubMed, and EMBASE by using the keywords ‘breast cancer’ or ‘BC’, ‘micro RNAs’ or ‘miRNAs’, ‘tumor suppressor miRNA’ or ‘TS miRNA’, ‘breast cancer signaling pathways’, ‘miRNAs as therapeutic target’, and ‘nanoformulations for TS miRNA delivery in BC cells’.

## 2. Tumor Suppressor miRNAs in BC

The miRNA substitution is an innovative method for examining tumor suppressors’ therapeutic effects. Since they are much smaller than proteins, require merely the entrance into the cytoplasm of their target cells to become active, and may be administered systemically using siRNA delivery methods and technologies, miRNAs offer a fresh possibility [19]. Therefore, the transport barrier for miRNA duplicates appears lower than that for DNA that codes for proteins [20]. Cancer-associated fibroblasts (CAFs) in the TME have higher amounts of TS miRNA, substantially reducing their ability to increase, migrate, and expand tumors [21].

### 2.1. Mir-125 a, b

MiR-125a and miR-125b are downregulated in HER2-amplification, and HER2-overexpression breast cancers [22]. Overexpression of these miRNAs decreases HER3 and HER2 mRNA and protein levels in SKBR-3 cells (a HER2-dependent cancer cell line of human mammary tissues), resulting in cell migration, intrusiveness, and decreased anchorage-dependent growth. This effect is minor in the case of non-transformed HER2-independent BC [23].

### 2.2. Let-7

Researchers looked at miRNA overexpression in distinguished and self-renewing cells from cell lines of cancerous breast cells. In a recent study, they discovered that expression of let-7 was much lower in BT-ICs (mammary tumor initiating cells) and increased with differentiation. Incorporating let-7 into BT-ICs stopped them from increasing, forming a mammosphere, or developing tumors and metastasis in vivo [24,25]. Overexpression of let-7 suppressed the expression of known cancerous targets, such as HMGA2 and H-RAS. By pulling down H-RAS, self-renewal was decreased but there was zero effect on differentiation in the BT-IC-enriched cell line.

In contrast, knocking down HMGA2 increased differentiation but did not affect self-renewal. Their findings showed that let-7 controls several BT-IC cell characteristics, and that let-7 offers a novel opportunity to target tumor stem cells with therapeutic RNA. Entry of the let-7 miRNA into tumors could decrease stem cells by stimulating proliferation and differentiation. RKIP (Raf kinase inhibitory protein) inhibits NF-κB signaling pathways and the MAPK-G protein-coupled receptor kinase-2. It inhibited bone metastasis and BC cell intravasation in a mouse model while growing expression of let-7 in BC cells. As a result, a chromatin-remodeling protein, such as HMGA-2, that activates pro-metastatic and pro-invasive genes like a snail, showed lower expression.

### 2.3. miR 31

MiR-31 has been shown to decrease the expression of pro-metastatic genes, preventing metastasis at many stages. The amount of miR-31 expressed by all normal breast cells is related to the tumor’s metastatic status. In non-malignant BC cells, it is observed in low amounts, reduced, and almost unnoticeable in human BC cell lines and metastatic mice. Moreover, introducing miR-31 in metastatic BC cells decreased in vivo and in vitro metastasis-related behaviors (resistance to anoikis, invasion, and motility) [26]. Even though miR-31 overexpressing mammary cancer cells produced more proliferative tumors, they were better enclosed and hence less invasive, demonstrating that miR-31 prevents early stages metastasis in case of disease. After being injected directly into the blood circulation, miR-31 overexpressing cells could not survive, demonstrating that miR-31 limits metastasis at various stages of the metastatic process. In the in vivo setting, inhibiting miR-31 activity increased metastasis and invasiveness. Frizzled3 (FZD3), myosin phosphatase rho interacting protein (M-RIP), matrix metallopeptidase 16 (MMP16) family member of ras homolog gene A (RhoA), integrin alpha-5 (ITGA5), and radixin (RDX) are six sigma aims of miR-31 in breast oncology. As miR-31 prevents metastasis by targeting several pro-metastatic human genes in a metastatic cascade, it undoubtedly has remarkable therapeutic promise for treating human BC.

### 2.4. miRNA-34a

The mechanism by which microRNA 34a (miR-34a) decreases cell growth is unknown, but miR-34a stops SIRT-1 from being expressed. A binding site of miR-34a exists in SIRT1’s 3 UTR. SIRT1 inhibition raises acetylated p53 levels, and PUMA and p21 are p53 transcriptional targets. These proteins participate in cell cycle processes as well as in apoptosis. In addition, inhibiting SIRT1 with miR-34 causes apoptosis in WT cells. However, p53 is missing from human colon cancer cells. Finally, the transcriptional target of p53 is miR-34a. A feedback mechanism could exist between miR-34a and p53. MiR-34a serves as an inhibitor in this way [27]. 

### 2.5. miR-200

One of mammals’ most well-known miRNA families is the miR-200 group, which comprises miR-429, miR-200b, and miR-200a. They play a vital role in EMT, drug resistance, cell proliferation, and other biological processes. The miR-200 family of microRNAs has been dysregulated in lung, ovarian, and stomach cancers, as well as BC.

The Wnt/β-catenin pathway is important for BCSC stability and regulates mammary cell growth at various stages. The miRNAs such as miR-200c/141, miR-29b, and miR-600 regulate metastasis, progression, and therapy resistant Wnt/β-catenin-mediated self-renewal in BCSCs. As a result, the miRNA-catenin and Wnt axis can be evaluated as a great target in developing an effective BC treatment. Wnt/catenin was activated when the miR-200c/141 cluster was depleted, suggesting that miR-141/200c regulated BCSC proliferation and formation through modulating catenin/Wnt signaling [26,27].

### 2.6. miR-145

MicroRNAs are key gene regulators that can have an impact on cancer. MiR-145 is a tumor suppressor that prevents tumor cell proliferation both in vivo and in vitro. MiR-145 is a cellular microRNA with a specialized function. However, miR-145 prevents cell proliferation in HCT 116 and MCF-7 cells and does not affect metastatic mammary cancerous cell lines. However, miR-145 inhibits cell invasion in these cells, whereas miR-145 against the antisense oligo improves the cell invasion process. MiR-145 has been shown to reduce lung cell metastasis in an experimental metastatic animal model, and miR145 prevents step-by-step cell invasion by inhibiting the metastasis process of gene mucin 1 (MUC1). Researchers recognized MUC1 functions as a direct target for miR-145 by implementing luciferase reporters having the 3′-untranslated part of MUC1 along with immunofluorescence labeling and western blot. In addition, ectopic MUC1 expressions stimulate the cell invasion process, which miR-145 inhibits. MiR-145 inhibits MUC1 and causes a reduction in β-catenin and the oncogenic cadherin11. Finally, RNAi inhibition of MUC1 imitates miR-145’s invasion suppressive action, which is connected to β-catenin and cadherin11 downregulation. This information shows that the role of miR-145 is as a tumor suppressor, limiting tumor growth, cell invasion, and metastasis [28].

### 2.7. miR-335

MiR-335 prevents the metastatic cell invasion process and controls the expression of a collection of genes related to the danger of distal metastasis in various human tumors. By specifically targeting the extracellular matrix component tenascin C and progenitor cellular transcription factors, such as SOX4, miR-335 inhibits metastasis and migration. Most relapsed patients’ original breast tumors lose miR-335 expression, and loss of microRNA is connected to deficient distal metastasis-free existence. Thus, miR-335 has been reported as a metastatic suppressor micro-RNA in human mammary cancer cells [29].

MiRNAs can directly target the ER, resulting in an advanced phenotype with endocrine resistance. While miR-335-5p is usually considered a tumor suppressor, it represses ER in mammary cancer cells. It is increased by estrogen activation, implying that it could be engaged in estrogen signaling alterations. MiR-335 expression was greater in normal cells than in tumor cells, identifying miR-335 as a tumor cell suppressor. MiR-335 transcript-directed expression on an ER+ BC cell line indicated that two strands of miR-335 double helix conveyed across mammary cancer cell lines with no evident link to clinical tumor subtypes. The expression vector of a pre-miR-335 was firmly introduced into the parental MCF-7 cell line, and qPCR was used to show that the cell line of MCF-7-miR-335 had overexpression of miR-335-5p. MiR-335-5p and miR-335-3p from the cell line of MCF-7-miR-335 were overexpressed, with intact 5-p and 3-p miRNAs. Finally, miR-335-5p and miR-335-3p can modify the RAC1-activity, CDH1 stability, ER signaling, and PDGFR signaling network based on suppressed gene sets [30]. 

### 2.8. miR-203

MiR-203 is expressed more in triple positive carcinoma cells, suggesting that these miRNAs may limit tumor growth in ER-positive BC. Overexpression of miR-203 decreased BT474 cell growth considerably, but antisense-mediated gene silencing repression of miR-203 massively increases cell proliferation. In addition, miR-203 prevents the growth of mammary cancer cells. Overexpression of miR-203 reduced BT474 cells’ migratory potential and invasiveness, but antisense-mediated reduction of miR-203 enhanced migration invasiveness and intensity, showing that miR-203 suppresses mammary cancer cell invasion and migration. Overexpression of miR-203 boosted cell cycle inhibitors p27 and p21, reduced cell-cycle activators CDK6 and cyclin D2, and increased apoptosis-associated protein Bcl 2 levels. Inhibition of miR-203 reduced the number of cell cycle inhibitors p27 and p21, elevated cell-cycle activators CDK6 and cyclinD2, and inhibited the apoptosis-related protein Bcl-2, corroborating previous findings. These studies examined the molecular mechanism of miR-203-induced mammary cell growth arrest. The amounts of MMP mRNA in miR-203-modified human mammary cancer cells were studied. Researchers discovered that miR-203 suppressed matrix metalloproteinase MMP7, 2 (MMP2), and MMP9 in BC cells but not other MMPs. Thus, the primary target is to study the inhibition of miR-203-mediated mammary cell invasion [31].

### 2.9. miRNA-339-5p

The transcription factor called the p53 tumor suppressor, which offers a framework for several stress-sensing pathways, is crucial for the cellular reply to oxidative stress. MiRNAs are 20–24 nucleotide short non-coding RNAs that affect several biological processes, including the regulation of p53. MicroRNAs (miRNAs) can either increase or decrease tumor growth. They control numerous important cancer-related pathways [32]. Thus, it has been discovered that miRNA dysregulation is a common trait in several human cancers. The tumor suppressor gene p53, which slows cell development in response to stress, is the one that is deleted and changed most frequently in human cancers. The oncoprotein MDM2 prevents p53 from working properly. The researcher, using a high throughput screening approach, found that, miR-339-5p acts as a p53 pathway regulator. By targeting the 3′-untranslated area of the MDM2 mRNA, miR-339-5p enhances p53 activity by lowering MDM2 levels [33]. Therefore, miR-339-5p overexpression encourages p53-regulated cellular actions such as stoppage of proliferation and cell death, whereas miR-339-5p inhibition inhibits the response of p53 in cancer cells, enabling enhanced proliferation [34]. Additionally, miR-339-5p levels are lower in tumors with wild-type TP53, indicating that lowering miR-339-5p levels decreases the p53 response in p53-competent tumor cells. Additionally, we demonstrate a negative correlation between MDM2 and miR-339-5p expression in human cancer, demonstrating the significance of the interaction for cancer [35].

### 2.10. miRNA-433

As per the research done in 2020 by Jinhui Xue et al., the existence speed of the high-expression group was much higher than that of the low-expression group. This result raised the possibility that miRNA-433 could be a marker for the malignancy of mammary cancer [36]. Shiqin Liu et al. initially argued that miRNA-433 could decrease tumor growth in ER+ BC by preventing M2 macrophage polarisation. The results of these investigations have shown that miRNA-433 deserves attention, has significant research value, and could open up a new way to treat breast cancer targeting miRNA-433 [37].

## 3. Challenges Associated with Traditional BC Therapies

**Figure 2:**
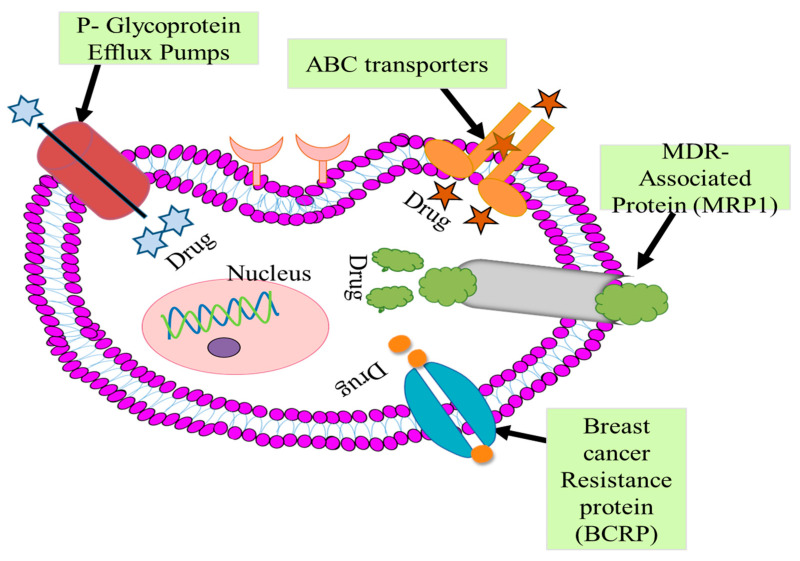
An overview of various transporter proteins: P-glycoprotein, ABC transporter, MDR-associated protein (MRP1), and breast cancer resistance protein (BCRP) are transporter for lipids, proteins, toxins, and sterols. These transporters show broad drug specificity and transport various anticancer drugs, causing lowering drug concentrations in tumor cells and thus reducing drug efficacy. All these transporters contribute to resistance to cancer chemotherapy.

MiRNAs have been reported widely to contribute to the response or resilience of cancer to intervention throughout the past ten years, including chemotherapy [38], radiation [39], hormone therapy [40], and immunotherapy [41]. Surgery, radiation, and chemotherapy are typical of the conventional therapies used to cure all kinds of BC. Surgery is sufficient to remove solid tumors in their early stages, but since it cannot kill all cancer cells it must be combined with chemotherapy and radiotherapy [42,43]. In contrast, chemotherapy has several drawbacks because it is effective against cancer cells but also causes damage to healthy cells [44]. Additionally, due to the high heterogeneity of tumor cells, multidrug resistance (MDR) may be present, which can cause metastasis and recurrence in patients [45]. Some challenges are shown in Figure 2. However, radiotherapy is appropriate for tumors in a specific location of the patient’s body and uses radiation to cause DNA damage-induced cell death. It is similar to chemotherapy, which may also result in non-cancerous cell death and recurrence. Current therapies try to address intra-tumoral heterogeneity and are site-directed [46,47].

### 3.1. P-Glycoprotein Efflux Pumps

PGP has been discovered in several tissues and was identified as an overexpressed protein in some multidrug resistance (MDR) BC cell lines. It removes hydrophobic or cationic and neutral compounds from cells (etoposide, vincristine, vinblastine, doxorubicin, paclitaxel, and daunorubicin). When PGP is used for transportation, the drug is taken directly from the lipid bilayer’s cytoplasmic side [48]. Many PGP substrates quickly separate into lipids and plasma membranes and are necessary for medication-stimulated ATPase activity [49].

### 3.2. ABC-Transporter

ABC transporters (ATP-binding cassette) transporters are trans-membrane transporter proteins discovered in drug-resistant cancer cells [50]. The primary ABC transporters linked to the development of multidrug resistance in mammary cancer types are many drug resistance-related BC resistance proteins (BCRP), P-glycoprotein (PGP), and protein-1 (MRP1) [51,52].

### 3.3. MDR-Associated Protein (MRP1)

#### Multidrug Resistance

BC treatment is accompanied by a significant problem of resistance to chemotherapy [53]. Many originally susceptible cancers have relapsed and developed resistance to various anticancer drug agents with various mechanisms of action and structures [54]. This is called multidrug resistance (MDR). The resistance mechanism is unknown; however, it may be related to the role of these drug-resistant transporter genes. A novel treatment is being developed to deal with drug resistance. To achieve therapeutic efficacy, chemotherapeutic resistance pathways must be well understood. A variety of factors can contribute to drug resistance [55]. This may be due to increased ATP-dependent enzyme activity. Drug concentrations within cells are reduced mainly by efflux pumps. Paclitaxel, doxorubicin, vincristine, daunorubicin, and vinblastine are all commonly linked to this resistance. A decrease in cellular drug levels can also cause resistance. The initiation of controlled detoxification systems like cytochrome-P450 oxidases enzymes and enhanced DNA repair is another major pathway of resistance.

### 3.4. Breast Cancer Resistance Protein

BCRP expression has been associated with developing resistance against anticancer drugs including mitoxantrone, camptothecins, anthracyclines, flavopiridol, and anti-folates. However, the mechanism of BCRP-assisted drug transport has not been examined as completely as that of PGP and MRP1. Hypoxia 27 increases the synthesis of BCRP, which may protect stem cells and tumor cells from anticancer drugs [56].

### 3.5. Microtubule Alteration 

Paclitaxel interacts with the tubulin subunit in microtubules, preventing dynamic instability and cell death. Overexpression of these subunits has been identified in clinical trials as a prospective biomarker for BC drug resistance [57]. Various tubulin mutations that provide pharmacological resistance to anti-microtubule medicines have been found in vitro [58].

This is one way that nanomedicine, a multidisciplinary science that includes fields like chemistry, molecular biology, medicine, and engineering, has significantly impacted the scientific community [41]. It allows the formation of vehicles made of nanomaterials (such as lipids, gold, polymers, metals, RNA nano-structure, silica, carbon, etc.) that can deliver their load through genes or CRISPR/Cas9, epigenetic regulators, and chemotherapeutic agents [59].

## 4. In Vivo Studies of Various Delivery Platforms 

The delivery techniques of miRNA are classified as either local or systemic. There have been studies on a variety of local delivery techniques, from surface-modified nanomedicines to direct intratumoral miRNA parenteral [60]. MiRNAs supplied locally can achieve the requisite gene silence because of their higher bioavailability. Some siRNAs can be delivered intratumorally as miRNA antagonists or inducers for cancer treatment.

Designing systemic miRNA delivery systems has made significant progress in overcoming the obstacles of in vivo miRNA delivery. The first strategy involves creating chemically changed miRNAs or antagonists like anti-miRNA oligonucleotides (AMOs) [61]. The second delivery strategy depends on the increased retention (EPR) effect and permeability and is used to create a nanomedicine for passive transfer through tumor tissues. Surface modifications to nanoparticles have recently emerged as a third-generation delivery method that allows specific binding to cancer cells [62]. Inorganic nanoparticles are non-viral, nonimmunogenic, and nontoxic delivery systems for miRNAs. AuNP-S-polyethylene glycol showed low toxicity, more miRNA loading capacity, and effective endosomal carrier release in human cancer cells. MiR-34a is selectively delivered to neuroblastoma cells by disialoganglioside GD2 (GD2) antibody-modified silica nanoparticles. The systemic administration of these nanoparticles resulted in greater apoptosis and a decrease in tumor vascular density [63,64]. Table 1 compiled of in vivo studies that investigated miRNA-based therapies for tumor cells.

## 5. Nanotechnology Enabled the Delivery of TS miRNAs in BC

In addition to reducing the likelihood of inactivation or degradation, nanoformulations can increase the spatiotemporal specificity of relatively unstable microRNA conjugates by prolonged circulation times and enabling targeted accumulation. The therapeutic potential of these nanocarriers is appealing due to their biocompatibility [69,70]. It is known that miRNA has a negative charge and is water soluble, which impedes its uptakes by cells, resulting in poor targeting capability, short half-life, and low stability [71]. To overcome these issues, miRNA must be modified into carrier systems [72]. The nanoparticle system is one such carrier that can help deliver the miRNA effectively. Nanoparticles offer unique ways to deliver the miRNA to controlled cells and produce required therapeutic actions [73]. Nanoparticles with sizes ranging from 60nm to 160nm, combined with miRNA, can treat diseases like cancer, diabetes, neurodegenerative diseases, and tissue regeneration [74].

Additionally, the formulation of nanoparticles with miRNA enhanced the in vivo delivery of the miRNA to the cells [75]. Moreover, as miRNA is miscible with water, miRNA’s diffusivity into the water phase is diffused when emulsion-based or nanoprecipitation methods are used to prepare the nanoparticles, leading to poor entrapment efficiency [76]. Therefore, modifications of the nanoparticles using ligands are preferred to enhance the uptake of the nanoparticles by receptor-mediated endocytosis, which decreases the dosage and the side effects caused by the formulation [77]. Another nanoparticle carrier that delivers miRNAs are mesoporous silica nanoparticles (MSN) with 2–50 nm pore sizes. These nanoparticles have been used to deliver siRNA and miRNA simultaneously. The MSN is loaded with the indocyanine green photosensitizer to enhance the penetration into tumor cells. Indocyanine green is irradiated by light, going towards the reactive oxygen species generation, ultimately releasing miRNA and siRNA from the formulation [78]. Thus, the delivery of the two different therapeutic RNAs leads to effective treatment against cancer.

Moreover, the formulation exhibited a higher rate of cellular uptake in both in vivo and in vitro experimental studies. Additionally, upon irradiation with short light, there was a significant reduction in tumor metastasis and the suppression of tumor growth in the primary lesion [79]. In one study, nanoparticles loaded with miR-34a and doxorubicin targeted the hyaluronic acid receptor, which exhibited effective tumor inhibition in the in vivo experiment [80]. Similarly, nanoparticles loaded with miR-18a and further modified with arginine-PEI demonstrated around a 91% reduction in the tumor volume and ATMK gene, resulting in reduced tumor growth. Nanoparticles loaded with paclitaxel and miR-34a, targeted against the Bcl-2 protein, exhibited the chemosensitization of the paclitaxel in cancer treatment. As mentioned earlier, nanoparticles are associated with gold metal ions. One such gold nanoparticle was loaded with the miRNA, which enhanced the absorption of the miRNA around 20 times when compared with the miRNA with lipofectamine with less toxicity and an enhanced half-life [81]. Some other cancers that are treated with nanoparticles associated with miRNA are pancreatic [82], lung [83], brain [84], and ovarian cancer [85].

**Figure 3:**
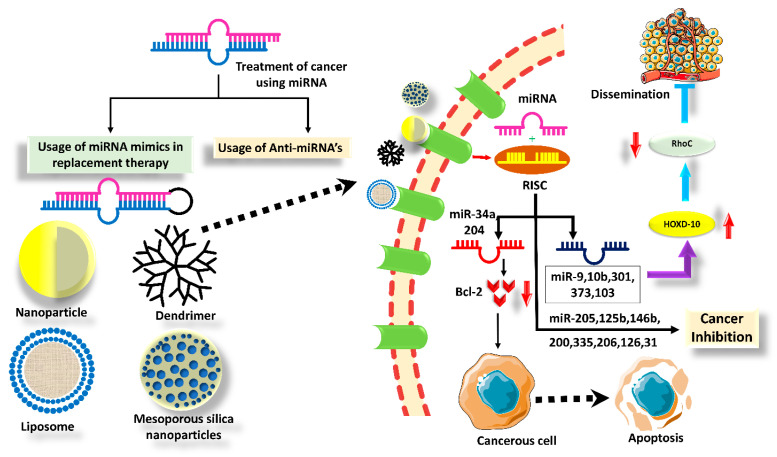
An overview of various delivery platforms through which miRNAs can be delivered to biological systems. Delivering tumor suppressor miRNA to primary tumor cells via nanoformulations. The optimum nanoformulation for TS miRNA should be well tolerated, safe, and capable of targeting tumors with few unwanted side effects. It should also be readily absorbed by cancer cells and deliver high concentrations of TS miRNA. This figure shows the delivery system for TS miRNAs to the cancerous cells and associated outcomes.

As shown in Figure 3, various ways of therapeutic miRNA delivery can also help detect miRNA in living cells. Occasionally, nanoparticles are associated with some metal ions like ferrous or gold metal ions. These metal ions allow the nanoparticles to bind with other elements covalently. With this, nanoparticles are covalently associated with ferric oxide elements, which are further attached to the hairpin assembly to direct the fluorescence of the complex; the association is stabilized using SS-DNA, which can detect miRNA in living cells. The SS-DNA used in the preparation of the nanoparticles can reduce the non-specific adsorption and decrease the signal-to-noise ratio during miRNA detection in living cells. The indirect method of detecting the miRNA produced more accurate results when compared with the direct method of detecting miRNA. The indirect method of identifying miRNA in living cells proves that using nanoparticles in the diagnosis is fruitful [86].

### 5.1. PLGA Particles

The water intake is greater than the rate of hydrolysis during bulk erosion of PLGA. The whole PLGA polymer matrix is involved in the breakdown of the polymer, which causes it to become water soluble. The degradation products now diffuse out of the deteriorating substance and become water soluble. Drugs having a high degree of crystallinity degrade more slowly, whereas amorphous phases degrade more quickly due to their high water absorption. These variables alter the drug release profile by affecting the degradation time, ranging from a few weeks to years. This feature of PLGA offers significant flexibility in regulating the drug release from a drug delivery system with the modified release [87]. High lactide content PLGA grade benefits drug delivery systems designed for prolonged action. The semicrystalline poly L-lactide can degrade for one to two years, while amorphous PLGA can degrade for one to two months.

Additionally, using PLGA helps the formulation bypass lysosomal degradation [88]. Therefore, PLGA is used in formulations that effectively deliver miRNA to cancer cells. Nanoparticles made of PLGA polymer were used to encapsulate PNA probes of short length that can target miRNA-155 and exhibited superior loading and uniform size distribution compared with conventional PNA probes.

Additionally, when the formulation was given to mice, the tumor size was significantly reduced, which motivates using the PLGA polymer [89]. In triple-negative breast cancer (TNBC), PLGA nanoparticles were loaded with miR-34a and poly-L-lysine (PLL), which released the miR-34a into the cytosol, targeting CCND-1 and other receptors, leading to cell death [90]. PLGA nanoparticles are also used for additive effects in combination with other drugs. For example, PLGA nanoparticles loaded with orlistat, combined with antisense-miR-21, enhanced the death of apoptotic and cancer cells [91]. In another study, doxorubicin and miR-542-3p targeted TNBC, whereas hyaluronic acid targeted the receptors [92]. This formulation exhibited low cytotoxicity with enhanced cellular uptake, and the activation of the p53 protein was identified, which shows the formulation’s effectiveness [93]. 

### 5.2. Dendrimers

Dendrimers are branched polymers with symmetrical structures (branches originating from the core and diverging towards the end). The minimum number of branches arising from the center is two, considered first-generation, and for every generation the number of surface sites of the dendrimers will double. The unique feature of the dendrimers is the dendritic effect, as it depends on the size, nature, and degree of cooperativity between the surface functional groups of the dendrimers [94]. Dendrimers help in the delivery of therapeutic nucleic acids effectively. The controlled synthesis provides the required property to the dendrimers, which can help deliver the miRNA effectively. Even though many polymers can form the dendrimers, only a few polymers, including polyamidoamine (PAMAM), poly-L-Lysine (PLL), carbosilane, phosphorus-derived dendrimers, and polypropylenimine (PPI), were suitable for developing the dendrimers that effectively deliver the miRNA [95].

Additionally, as different polymers have different structures, they have different steric limitations, providing researchers with additional work choices. High-generation PAMAM dendrimers are the most widely used to deliver miRNA. The synthesis of core-shell tecto dendrimers (CSTDs) structurally resembles high-generation PAMAM dendrimers and exhibits the same properties. The CSTDs were prepared by joining cyclodextrin and adamantane by forming the supramolecular host guest. Such CSTD technology was used to prepare dendrimers encapsulating miRNA 21i (microRNA 21 inhibitor), which exhibited significant delivery of the miRNA to cancer cells, decreasing cancer cell migration and decreasing the miRNA-21 gene expression [96].

Additionally, the same dendrimers loaded with doxorubicin exhibited similar results. Dendrimers associated with miRNA were also used as help for the imaging of tumors. PEGylated BODIPY dyes and the miRNA were formulated into dendrimers used for the near-IR imaging of the cancer cells. Moreover, the formulation contained a pH-responsive lipid, which gives accuracy in imaging cancer in vitro and in vivo [97]. Additionally, dendrimers were also used for the diagnosis of the tumor. To achieve this, DNA-peptide dendrimers were combined using mass spectroscopy, i.e., RP8-MAP4-DNA, used to target miR-21 [98]. The probe was applied on three BC cell lines and tested on BC tissues, where the promising results led to a new way to use dendrimers in association with miRNA in the field of cancer therapeutics [99,100]. 

### 5.3. Polyethyleneimine (PEI)

PEI is a polymer with a linear or branched structure, dissolved in water, and is proved to be an excellent vector for the delivery of the miRNA [101]. The transfection efficiency of the vectors made with PEI polymer is good compared with other polymers [102]. However, PEI polymer has some limitations as it is cytotoxic on PM. For biocompatibility, a conjugation of the polymer with other chemicals is used, which produces the desired effects. An example of one such modification is the addition of N-isopropyl acrylamide to PEI25 K to produce a derivate named PEN, which has enhanced biocompatibility [103]. Delivering miRNA with the PEI polymer’s help is more efficient when conjugated with other molecules; one such linkage is the creation of the disulfide bond, cross-linked PEI. The disulfide-linked PEI is used to entrap anti-miR-155 in a nanocarrier, degraded by glutathione, leading to anti-miR-155 to reduce cancer growth [104]. As mentioned earlier, PEN, a derivative of PEI polymer, was used as a carrier for miR-29a, which induced apoptosis by arresting the cell cycle at the G1 phase, showing that this therapy might be useful as an effective cancer treatment [105,106]. 

### 5.4. Liposomes

Liposomes are drug delivery systems characterized by a double lipidic layer surrounding an inner water core that can actively and passively target BC cells. The ideal size range of liposomes is between 80 and 150 nm for cell uptake [107]. Liposomes can be directed to malignant tissue (EPR) through the improved permeability and retention effect. The method is further enhanced by targeting cancer cells by employing ligand- or antibody-mediated interactions. Several ligands, especially estrone, folate, and transferrin, have already been investigated for liposome-based targeted drug delivery to breast cancer. Additionally, monoclonal antibodies have been examined, and miRNA-7 has been shown to stop solid tumors’ growth, invasion, and spread. In a mouse xenograft ovarian cancer model, miR-7 surrounded by liposomes was successfully transferred to cancerous ovarian cells and targeted to the tumor location. Liposomes possessed the right particle size, potential, and cell absorption rate. Yan Yan et al. developed a special sense strand of miRNA of 25 nucleotides packaged inside the functional liposomes that were altered with the DSPE-PEG-2000-tLyp-1 peptide for treatment by silencing the slug gene [108]. These liposomes increased the anticancer efficiency of adjuvant chemotherapy in mice, prevented the TGF-1/Smad pathway, and decreased slug expression in triple-negative BC cells. The latest review thus revealed a promising way of using gene therapy to treat invasive human mammary cancer.

### 5.5. Modified Extracellular Vesicles (EVs)

TS miRNAs have been supplied via modified EVs from various cell sources, but MSC-EVs offer a favorable natural homing/immune evasion property. The metalloproteinase 15 (A15) and protein A disintegrin, which has good bonding for integrin v3, enable the targeting of the integrin v3 overexpressing malignancies like a severe case of triple-negative breast cancer (TNBC). When subjecting phorbol 12-myristate-13-acetate (PMA) to monocytes, additional vascular fluid is released that contains enough membrane-bound A15. It was shown that miR-159 reduced BC patient plasma levels and inhibited BC growth through the Wnt signaling pathway. To increase miRNA stability and cell internalization, the 30 ends of miR-159 were chemically modified to add hydrophobic cholesterol (Cho–miR-159). Cho-miR-159 and the chemotherapeutic drug DOX were incorporated within the nanoparticles targeted formulation (Co–A15–EV). A15-EVs were far more effective at targeting TNBC tumors than regular EVs, which dispersed off-target in the kidney and hepatic tissue, according to preliminary mouse biodistribution research. The co-delivery of miR-159 and DOX surrounded in A15-EVs produced the best treatment response, with 53 days average survival rate, compared with 27 days survival rate when mice were treated with saline [109]. Table 2 shows the different nanoparticles used to deliver TS miRNAs and respective associated cellular mechanisms.

### 5.6. Challenges in Nano Drug Delivery

Only a few nanoformulations have made it into clinical trials, indicating that developing effective cancer nanotherapeutics remains difficult. The physicochemical properties of nanoparticles influence their biocompatibility and toxicity. They show interactions with biomolecules and may accumulate, making a protein corona that disrupts the normal role of nanomedicine formulations and renders them non-effective. Due to unfavorable interactions with biological entities, nanocarriers in cancer treatment may cause unintended toxicity. Commercialization of nanomedicine products is also a significant challenge. Another significant challenge is obtaining regulatory approval for nanomedicines, as the FDA must establish specific criteria for products containing nanomaterials [116]. Successful miRNA distribution by nanoparticles faces some difficulties. First, when nanoprecipitation or emulsion-based preparation techniques are used, miRNAs rapidly diffuse into the aqueous phase due to their strong affinity for water, leading to a low encapsulation efficiency. The required dosage is minimized to promote uptake by receptor-mediated endocytosis, and the NP surface must be changed with ligands for targeting the target cells. It costs money to be biofunctionalized in this way. As miRNAs can decay in the lysosome’s low pH if released from NPs too late, another issue is the transfer of conjugated or encapsulating miRNA into the cytoplasm. Furthermore, the choice of the substance and the encapsulated miRNA loading procedure for a nanoparticle is of utmost importance. 

## 6. Advanced Strategies for TS miRNA Delivery in BC

### 6.1. Viral Delivery

Viral vectors were used to transfer pre-miR or mature miRNA to tumor cells after their cloning in a plasmid, which produce mature miRNA, boost its expression, and inhibit or degrade target mRNAs. MiRNA mimics or antagonists have been successfully delivered to tumor cell nuclei by lentivirus, adenovirus, and adeno-associated virus (AAV)-mediated delivery techniques, followed by miRNA expression and function [117]. In a New Zealand Black (NZB) mouse model of chronic lymphocytic leukemia (CLL), Kasar et al. showed that the lentiviral delivery of miR-15a/16 restored the expression of these miRNAs, whose expression was lost in CLL. In a different study using a mouse model of BC, it was discovered that intravenous administration of lentiviruses expressing miR-494 antagonists reduced the activity of myeloid-derived suppressor cells (MDSCs) by blocking miR-494, which is pro-angiogenesis and encourages tumor growth. Although functional miRNA antagonists or miRNA can be effectively delivered into tumor tissues via viral vectors, the immunogenic response is still a significant concern in clinical applications. As a safer alternative, many non-viral delivery techniques for miRNA have been created [118].

### 6.2. Self-Assembled RNA-Triple-Helix Hydrogel Drug Delivery System

The hydrophobic nature of cholesterol could confine the RNA-triple-helix hydrogel to a specific region; therefore, the diameters of the hydrogel ranged from 500 nm to 200 nm in diameter. Hydrophobic interactions between cholesterols also led to the formation of a hydrogel. Moreover, cholesterol enhanced in vivo transfection efficacy and shielded miRNA against deterioration. In addition, the cholesterols released their negative charge, and the RNA transcripts’ zeta potential was also increased. The molecular weights of RNA transcripts varied widely as well. Thus, the RNA transcripts and RNA-triple-helix hydrogel were characterized using gel electrophoresis. The varied self-assembled structures in the RNA-triple-helix hydrogel resulted in different electrophoretic mobilities. At a concentration of 1 mM, RNA-triple-helix hydrogel, miRNA-205, and miRNA-221 did not travel along the electrical gradient and remained in the well. Ding, Lairong, et al. created a new RNA-triple-helix hydrogel for TNBC detection and treatment using a programmed self-assembly technique. The RNA-triple-helix hydrogel offers benefits like greater biocompatibility, improved nuclease resistance, and effective cellular absorption. Combining aptamers and therapeutic genes into various building blocks, like RNA hydrogel, can also be employed in targeted and stimuli-responsive gene regulation treatment, stopping MDA-MB-231 cells from proliferating and migrating. Compared with unbound miRNA and RNA transcripts, higher in vivo absorption and in vitro selectivity are both displayed by the self-assembled RNA-triple-helix hydrogel, which also more effectively controls miRNA expression [119]. This highly selective gene delivery technology offers a potential targeted treatment for TNBCs. When combined with other miRNAs, triplex-helix hydrogel constructs can be produced and used to treat human cancers.

### 6.3. Hyaluronic Acid/Protamine Sulfate Interpolyelectrolyte Complexes (HP/IPECs) 

Nanocapsules based on hyaluronic acid/protamine sulfate interpolyelectrolyte complexes (HP/IPECs) have been designed for the encapsulation and intracellular delivery of miR-34a (BC tumor suppressor). It has been found that miR-34a encapsulated in HP/IPECs is effectively administered to BC cells or tissues both in vitro and in vivo. HP/IPECs targeted miR-34a to target CD44 and the Notch-1 signaling pathway, resulting in proliferation, migration reduction, and death induction [120].

## 7. Discussion

Analyzing the functional roles of miRNAs has been made possible because of the development of experimental techniques and bioinformatic tools. The results are promising, and miRNAs have great potential as next-generation therapies. However, more study is needed to determine the potential dangers of using miRNAs in vivo and their interactions with genes linked to BC. Before considering miRNAs as next-generation medication, issues including degradation by RNase in vivo and preventing unintended stimulation of an immune cascade must be handled. To consider miRNAs as viable biomarkers for accurately identifying the severity of BC, we need to identify the expression pattern of miRNAs linked to BC. Several sophisticated animal investigations and clinical trials are needed for miRNAs to qualify as therapeutic candidates [121]. The potential of miRNAs depends on efficient delivery to the target, the time of action, various concentrations, and the type of microenvironment. Safety, efficacy, targeted delivery systems, and optimized chemical modifications for miRNA as modulators are needed to solve these issues. The heterogeneity of BC may be the root of several inconsistencies between studies. According to this viewpoint, better patient inclusion criteria or a larger cohort size could eliminate the variations in miRNA expression caused by the disease’s basic heterogeneity. Since many pathological disorders (such as inflammation or cardiovascular diseases) and physiological circumstances, like food or physical activity, could significantly impact miRNA levels, patients’ therapy should also consider these factors. Hence, within a single investigation, the choice of internal controls should also be carefully assessed. Recent studies have a more precise emphasis on certain BC subtypes, like TNBC or HR+ BC. To reduce these previously mentioned potential interferences, they are performed in specific therapeutic contexts [122].

## 8. Future Prospects 

Since this review emphasizes tumor suppressor miRNAs, we mainly explain the methodologies of miRNA therapeutic delivery in the treatment of BC in a portion of miRNA-based therapy [123]. MiRNA can be delivered effectively to the cells using miRNA replacement therapy, where vectors are used as carriers to deliver effectively to the cancer cells. Future research should focus on NCOA4, initially identified as a coactivator of several nuclear hormone receptors and strongly associated with breast carcinogenesis and progression [124]. Thus, future combined therapy of ferritin autophagy regulated by cell ferroptosis and miRNA-mediated suppression of BC cells can be used for better BC management. These concepts require additional investigation through subsequent studies for convincing results [125]. As possible therapeutic targets for stopping epithelial–mesenchymal transition (EMT) to stop cancer spread, cancer-associated fibroblasts (CAFs) encourage EMT remolding [126]. Although they can treat regions of the body that other delivery systems cannot reach and deliver therapies, there are difficulties in clinically translating nanoparticle-based medication delivery for BC [127]. Nanotechnology-based approaches will unavoidably support future advances in customized medicine and pave the way for improved cooperation among professionals in clinical cancer, pharmacokinetics, toxicology, immunology, and nanotechnology. On a molecular level, miRNAs control numerous signaling pathways to regulate BC biological processes [128]. Therefore, miRNA profiling and comprehension are crucial for BC diagnosis, subtyping, prognosis, and recurrence and treatment response prediction. Additionally, numerous anti-oncomiRs and tumor suppressor miRNAs have been created as treatments that can be used alone or in conjunction with conventional BC therapy [129]. They can be discharged in liquid biopsy samples including urine, blood, milk, and other physiological fluids. Therefore, circulating miRNAs may one day serve as non-invasive biomarkers for BC care, including those for diagnostic, prognostic, and molecular subtyping, monitoring medication response, relapse prediction, and developing new therapeutic approaches. Examining the circulating miRNAs could provide molecular insights into BC’s origins, treatment resistance, and recurrence. It is possible to combine proteomics, genomics, metabolomics, and transcriptomics, to create multidimensional data. These data can be used to find unique circulating miRNAs in the liquid biopsy that have clinical relevance and utility in managing BC. These data can be integrated with different detection techniques and robust statistical analysis.

## 9. Conclusions

MiRNA is critical in oncogenesis, even though additional research on the molecular pathways underlying BC has been conducted over the years. There are still challenges in the initial assessment and treatment of women with BC, such as unanticipated responses and resistance to therapeutic compounds. As miRNA regulators, miRNAs may be used as innovative prognostic and diagnostic markers and new therapeutic checkpoints. The participation of miRNAs in the pathogenesis and maintenance of cancer development could be considered, making innovative improvements to existing cancer diagnosis and therapy methodologies. MiRNA therapy is found to be effective in the treatment of BC. The main challenge for the formulator arises with the effective delivery of the miRNA to the cancer cells. So, to deliver the miRNA effectively, it is encapsulated into various carriers like nanoparticles, dendrimers, and liposomes, which can deliver the miRNA to the cancer cells with the help of targeting. 

## Figures and Tables

**Table 1:** Examples of in vivo studies that investigated miRNA-based therapies to target tumor cells in preclinical animal BC models and other cell lines.

Sr. No.	miRNA	Delivery Systems	Cell Lines	Delivery Route In Vivo	Targeted Gene and Molecular Pathway	Ref.
1.	miR-200	(DOPC) nanoliposome	MCF 7, MB231	Inoculated subcutaneously	IL8, CL1XC	[65]
2.	miR-132	cRGD	HUVEC, MDA-MB-231, RCP30	Inoculated subcutaneously	p120RasGAP	[66]
3.	miR-542-3p	PEI-PLGA	MDA-MB-231, MCF 7	-	CD44, P53	[67]
4.	miR-4306	Lentivirus	MCF-7, T47D, ZR-75–1, SK-BR-3, HeLa, HCC1937, MDA- MB-468	Inoculated subcutaneously	VEGFA SIX1, Cdc42	[68]
5.	miR-155	PLGA	HCC1937, HIF1 RCP30	Inoculated subcutaneously	VHL	[69]
6.	miR-21, miR-145	Magnetic nanoparticles	MCF-7, HBL100	-	P53	[48]

**Table 2:** The potential therapeutic delivery of TS miRNAs in BC and associated mechanisms.

S. No	Therapeutics miRNA	Mechanism/Receptor Targeted	Nano-Vehicles Manufacturing Materials	Ref.
1.	miRNA-376b	Blocked autophagy	Superparamagnetic iron oxide nanoparticles	[110]
2.	miRNA-34a	Reduces BC cell migration, tumor growth, and proliferation	Polymers, gold, silica, liposomes	[111]
3.	miRNA-603	Reduction in angiogenesis and cell migration, proliferation, invasion, and tumor growth	Liposomes	[112]
4.	miRNA21	Decrease in tumor growth, cell proliferation, and migration	Graphene/polymer hybrids, chitosomes, polymers,	[113]
6.	miRNA-22-3p	Reduces invasion, tumor growth, colony formation, cell proliferation,	Lipids	[114]
7.	miRNA-200c	Reduction in the invasion, multidrug resistance EMT, cell motility	Polymer hybrids/peptide/lipid	[115]

## Data Availability

Not applicable.

## Abbreviations

BC—breast cancer; TS—tumor suppressor miRNAs; smiRNAs or miRs—microRNA; Akt—serine or threonine kinase, or BMI-1-B-lymphoma Mo-MLV insertion region 1; CBFB—core binding factor subunit beta; CSC—cancer stem cells; DNA—deoxyribonucleic acid; ERK—extracellular-regulated kinase; EMT—epithelial mesenchymal progress; HBXIP—hepatitis B X-interacting protein; EGFR—epidermal growth factor receptor; EMT—epithelial to mesenchymal transition; GPCR—G protein coupled receptors; ER—estrogen receptor; ERBB2/HER2—receptor tyrosine-protein kinase erbB2; HGF—hepatocyte growth factor; EPO—erythropoietin; FSCN1—fascin homolog 1; EPOR—erythropoietin receptor; HMGA2—high mobility group A 2; IGF-IR—insulin-like growth factor I receptor; KLF4—Krüppel-like factor 4; HUVEC—human umbilical vein endothelial cells; MMP 2—matrix metalloprotein 2; miRs or miRNAs—microRNAs; MMP 9—matrix metalloprotein 9; OCT-4—octamer-binding transcription factor-4; KRAS gene—Ki-ras2 Kirsten rat sarcoma viral oncogene homolog; MET—mesenchymal–epithelial transition; NK cells—natural killer cells; T-IC—tumor-initiating cells, UTR—untranslated region; PTQA—post-transcription quality articulating; RNA—ribonucleic acid; MAPK—mitogen-activated protein kinase; PDCD—pyruvate dehydrogenase complex deficiency; SOCS3—suppressor of cytokine signaling 3; RKIP—Raf kinase inhibitory protein; TS—tumor suppressor miRNAs; RISC—RNA induced silencing complex; RBP—RNA binding protein; TGF-β—transforming growth factor beta; TG—transglutamate; TS miRs—tumor suppressor miRNAs; ER+—estrogen-receptor positive; TNBC—triple-negative breast cancer; ZEB1—zinc finger E-box binding homeobox 2; VEGF—vascular endothelial development factor; ZEB1—zinc finger E-box binding homeobox 1; DOPC—dioleoylphosphatidylcholine; cRGD—arginyl-glycyl-aspartic acid (RGD); PEI—polyethyleneimine
